# Supplementary figures and images for: Using transcriptome sequencing (RNA-Seq) to screen genes involved in β-glucan biosynthesis and accumulation during oat seed development
Source: PeerJ. 2024 Sep 25;12:e17804. doi: 10.7717/peerj.17804 (PMC11438436; doi:10.7717/peerj.17804)

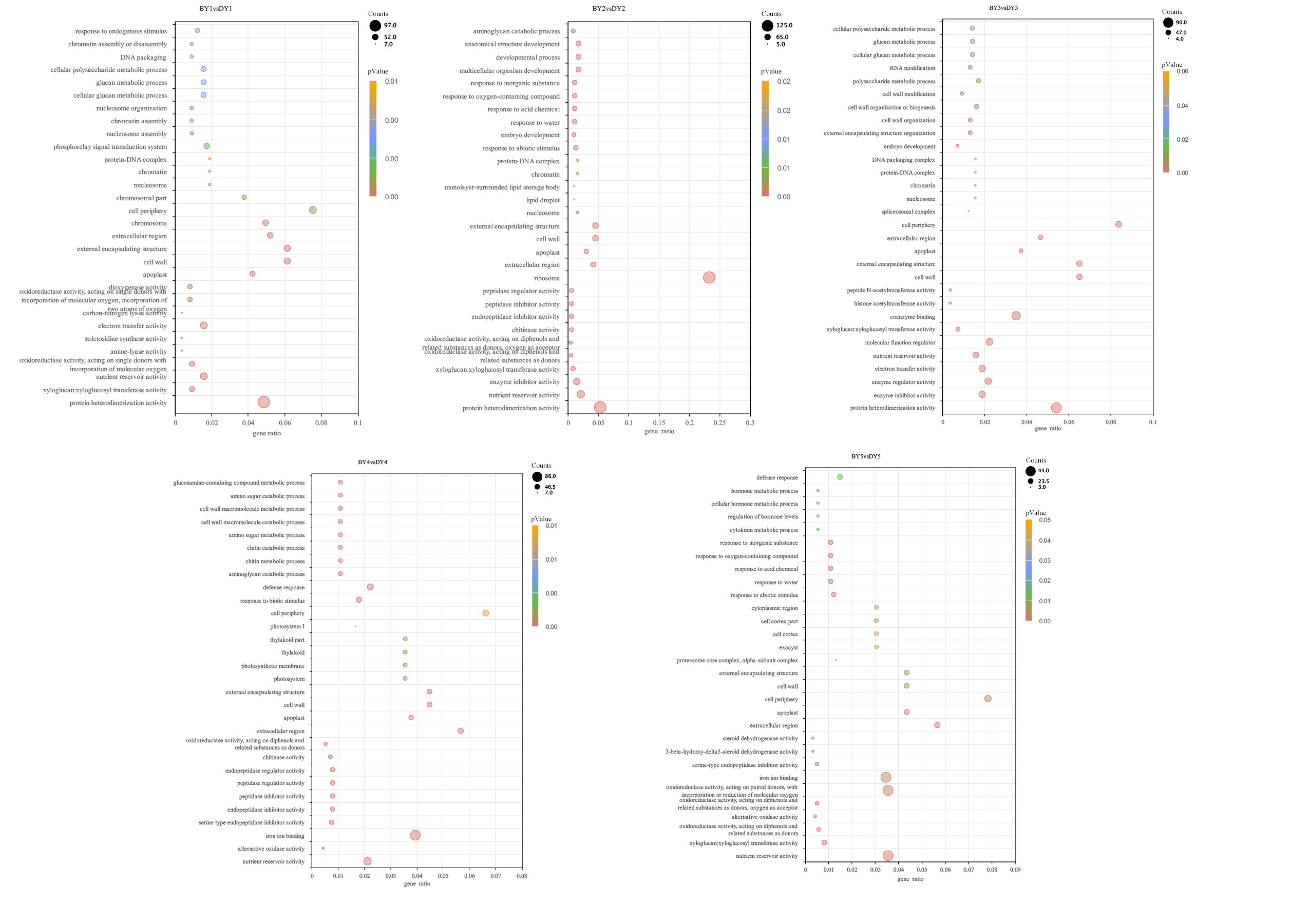

Supplement: Supplemental Information 1 — The color of the dot indicates PValue and the size of the dot indicates the number of differential genes enriched to that term. [file peerj-12-17804-s001.png]

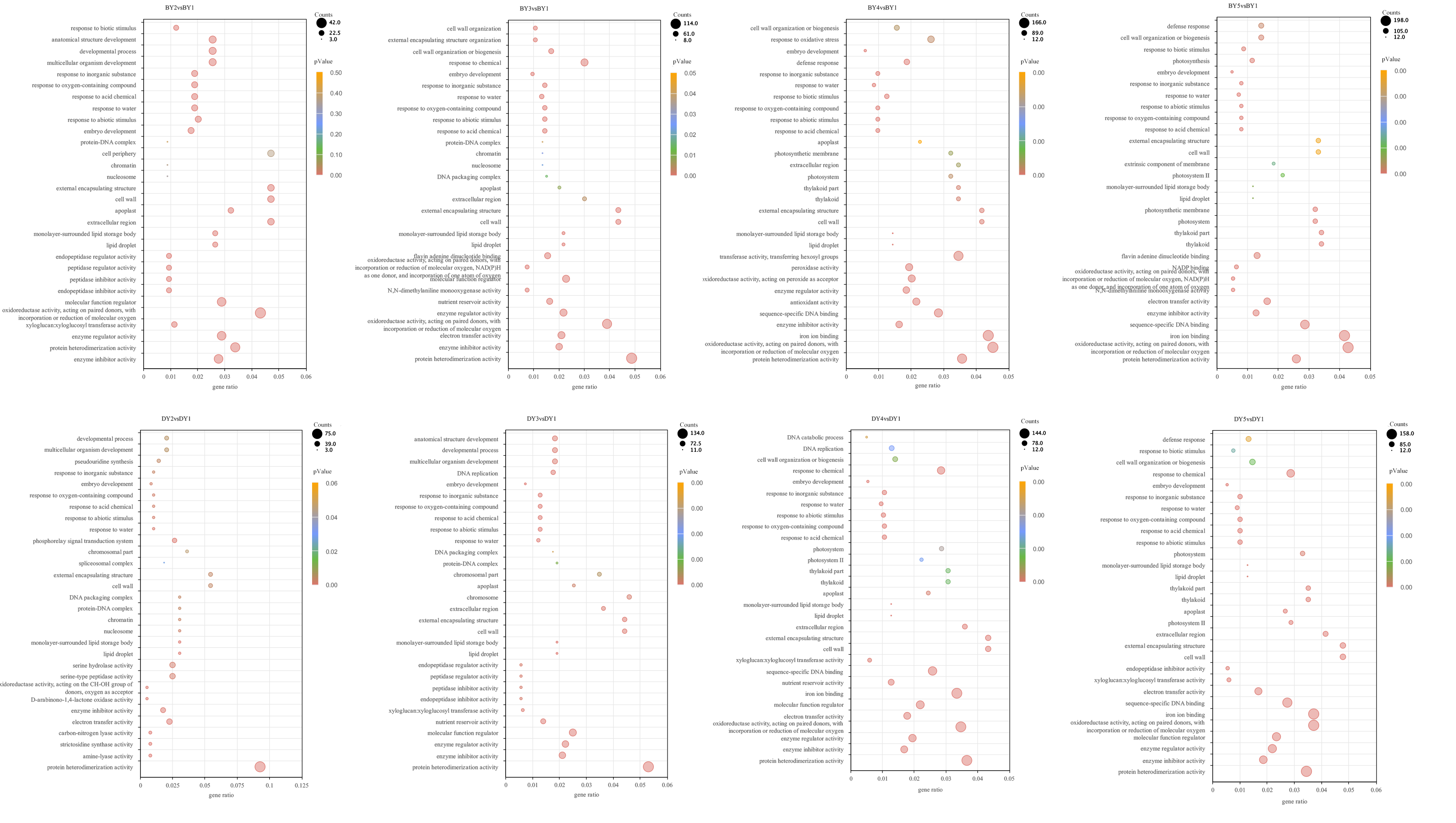

Supplement: Supplemental Information 2 — The color of the dot indicates PValue and the size of the dot indicates the number of differential genes enriched to that term. [file peerj-12-17804-s002.png]

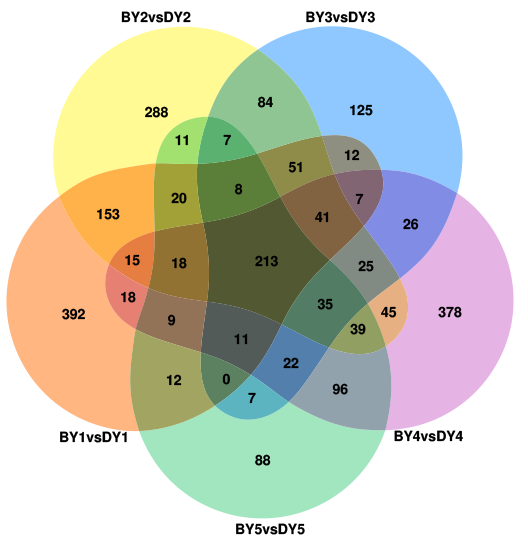

Supplement: Supplemental Information 3 [file peerj-12-17804-s003.png]

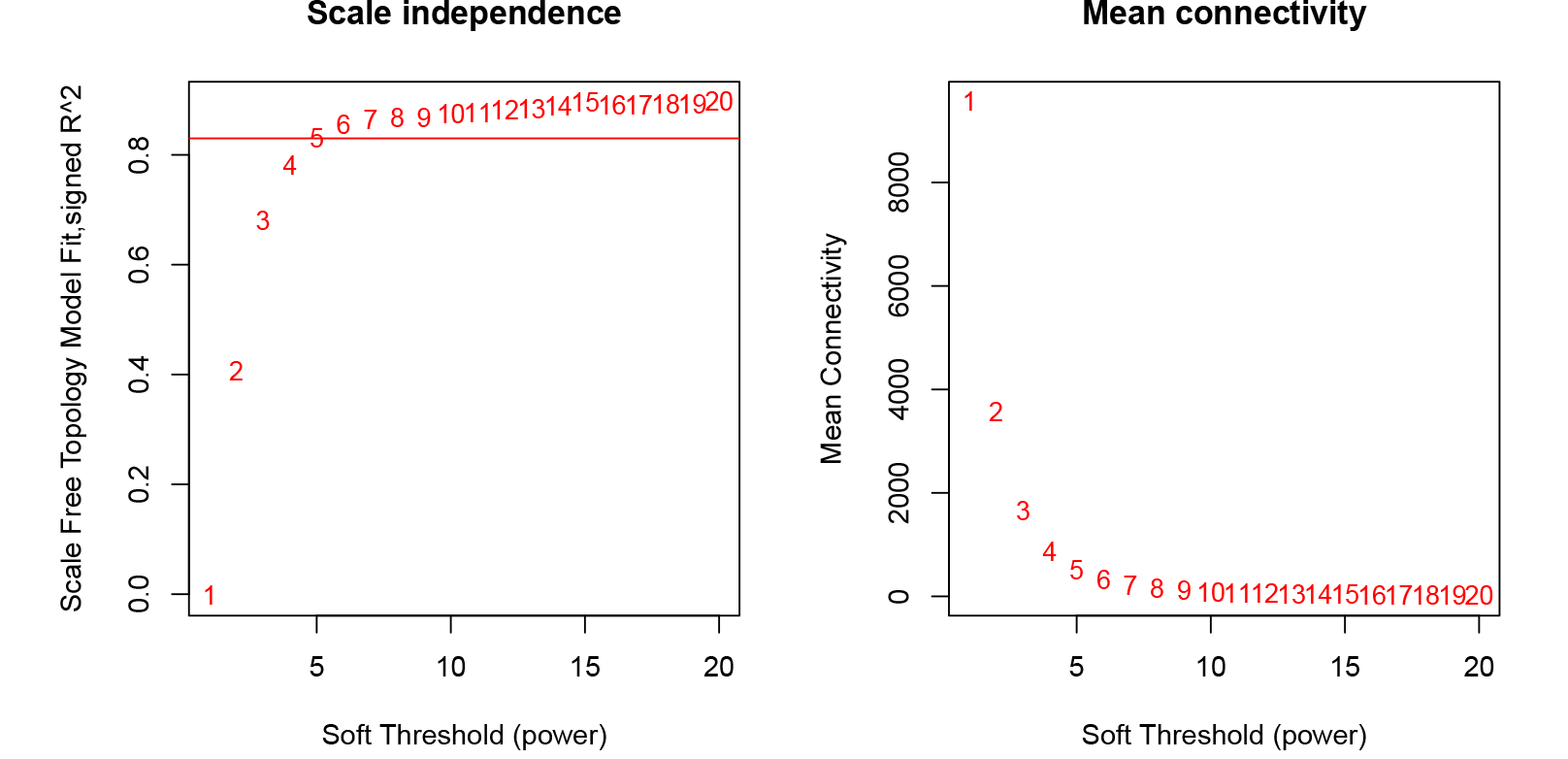

Supplement: Supplemental Information 4 [file peerj-12-17804-s004.png]
